# Effect of nutritional status before femoral neck fracture surgery on postoperative outcomes: a retrospective study

**DOI:** 10.1186/s12891-021-04913-2

**Published:** 2021-12-08

**Authors:** Katsuya Yokoyama, Taku Ukai, Masahiko Watanabe

**Affiliations:** 1 Department of Orthopedic Surgery, Tokai University School of Medicine Oiso Hospital, 21-1 Gekkyo, Oiso, Kanagawa 259-0198 Japan; 2 Department of Orthopedic Surgery, Surgical Science, Tokai University School of Medicine, 143 Shimokasuya, Isehara, Kanagawa 259-1193 Japan

**Keywords:** Femoral neck fracture, Geriatric nutritional risk index (GNRI), Nutritional status, Bipolar hemiarthroplasty (BHA)

## Abstract

**Background:**

Although nutritional status is crucial in gait recovery after femoral neck fracture surgery, the relationship between preoperative nutritional status and postoperative outcomes remains unknown. This study examined the effects of preoperative nutritional status on postoperative outcomes in patients undergoing femoral neck fracture surgery.

**Methods:**

Data regarding the joints of 137 patients (29 men, 108 women) who underwent bipolar hemiarthroplasty for femoral neck fractures at our hospital from January 2015 to December 2019 were retrospectively examined. The Geriatric Nutritional Risk Index (GNRI), an index of nutritional status, was used to classify patients into two groups: a normal group (GNRI ≥92; *n* = 62) and an undernourished group (GNRI < 92; *n* = 75). The study endpoints included age at surgery, sex, Mini Mental State Examination (MMSE), American Society of Anesthesiologists Physical Status (ASA) classification, preoperative waiting period, intraoperative blood loss, surgery time, perioperative hemoglobin levels, blood transfusion rate, complication rate, 6-month mortality rate, transfer rate, percentage of patients unable to walk at discharge or transfer, and inability to walk 6 months postoperatively.

**Results:**

The patients in the undernourished group was significantly older at surgery (*p* < 0.01) and had a lower perioperative hemoglobin levels (*p* < 0.01), a higher blood transfusion rate (p < 0.01), a lower MMSE (p < 0.01), a longer preoperative waiting period (*p* < 0.05), a higher transfer rate (*p* < 0.05), were more likely to be unable to walk 6 months postoperatively (*p* < 0.01), a higher complication rate (p < 0.05), and a higher 6-month mortality rate (p < 0.01) than the normal group. Patients in the undernourished group had worse rates of postoperative complications, transfer, mortality, and inability to walk 6-month after surgery than those in the normal group.

**Conclusions:**

A poor nutritional status affects the gait function and systemic condition of patients undergoing femoral neck fracture surgery; therefore, early nutritional interventions may reduce mortality rates and shorten rehabilitation. These results suggest that the GNRI effectively predicts postoperative complications, mortality, and gait function.

## Background

Proximal femoral fractures have become increasingly common, affecting nearly 1.5 million people worldwide each year. As the population ages, the annual number of femoral fractures is predicted to increase to 6.3 million by 2050 [1–4]. With a 1-year mortality rate of 20 − 30%, proximal femoral fractures are considered serious osteoporotic fractures [4, 5]. A study found that delaying surgery for proximal femoral fractures by more than 4 days significantly increased mortality [6]. Proximal femoral fractures may lead to gait disturbances and impaired activities of daily living (ADLs). Therefore, predicting the risk factors for poor postoperative outcomes and the recovery of gait function is crucial for the aging population [7].

Undernutrition has been associated with exacerbating complications following proximal femoral fracture surgery as undernourished patients are less likely to be discharged home due to deterioration in their systemic condition [8]. In current practice, undernourished patients are being screened preoperatively using several nutrition assessments that predict complications and mortality [1, 9]. The Geriatric Nutritional Risk Index (GNRI) [10] was used to determine the patients’ nutritional status in this study. GNRI is a simple index of nutritional status that can be calculated using only the serum albumin level, height, and body weight and that can be assessed at admission.

Although nutritional status is crucial in gait recovery after proximal femoral fracture surgery, the associations between preoperative nutritional status and postoperative outcomes are unknown. In the present study, nutrition status was assessed using the GNRI, and the effects of the preoperative nutritional status on postoperative outcomes were evaluated in patients who underwent treatment for femoral neck fractures at our hospital.

## Methods

### Patients

Data of 137 joints in 137 patients (29 men; 108 women) who underwent bipolar hemiarthroplasty (BHA) at our hospital for femoral neck fractures from January 2015 to December 2019 were retrospectively reviewed to assess nutrition status using the GNRI. This study was approved by the appropriate institutional review board (approval no.: 21R-042).

Patients with at least 6 months of follow-up data were included in this study. Patients for whom preoperative body weight, height, and serum albumin data were not available were not included in the study. In addition, patients who required wheelchairs or were bedridden before the surgery and those who underwent BHA for nonunion or osteonecrosis of the femoral head following osteosynthesis were also excluded from the study.

### Nutritional index

The GNRI is a simple index developed by Bouillanne et al. that can be calculated using serum albumin, height, and body weight (GNRI = 1.489 × serum albumin (g/L) + 41.7 × (weight/ideal weight)) [10]. In this study, the ideal weight was calculated using the Lorenz equations (men: height (cm) – 100 – [(height (cm) – 150)/4]; women: height (cm) – 100 – [(height (cm) – 150)/2.5]) [10]. Values for serum albumin and body weight were obtained on the day of admission.

A GNRI cutoff value of 92 was used to classify the patients into a normal group (GNRI ≥92; *n* = 62) and an undernourished group (GNRI < 92; *n* = 75), as previously reported [9–11].

### Endpoints

First, we evaluated the correlation between each patient’s surgical age and GNRI. Second, the patients’ age at surgery, sex, the preoperative waiting period, intraoperative bleeding volume, surgical time, the American Society of Anesthesiologists Physical Status (ASA) classification [12], Mini Mental State Examination (MMSE) [13], pre and postoperative hemoglobin levels, blood transfusion rate, complication rate, 6-month mortality, transfer rate, percentage of patients unable to walk at discharge or transfer, and inability to walk 6 months postoperatively were used as study endpoints. Two weeks postoperatively, patients were transferred to the rehabilitation facility if discharging them home was deemed too difficult. The severity of medical conditions at the time of surgery was evaluated by an anesthesiologist using the ASA classification.

### Functional outcome

The Patients’ ADLs were evaluated in 5 stages: 1: independent; 2: 1 cane; 3: walker; 4: walking along; 5: a wheelchair or bedridden. At the time of admission, the ADLs of patients before femoral neck fracture were evaluated, and the ADLs of patients 6 months postoperatively were evaluated. All data on ADLs were obtained from the patients’ medical records.

### Surgery

All patients underwent BHA via the posterior approach while in a lateral position. After the short external rotators and posterior capsule were incised and the femoral head was removed, a cementless system was implanted. The short external rotators and posterior capsule were then sutured to the greater trochanter. During rehabilitation, full weight-bearing was permitted, starting on the day following surgery.

### Statistical analyses

All statistical analyses were performed using IBM SPSS Statistics (version 26; IBM, Corp., Armonk, NY, USA). The Student’s *t*-test was used to assess age at surgery, the preoperative waiting period, MMSE, pre and postoperative hemoglobin levels, intraoperative bleeding volume, and surgical time. Fisher’s exact test was used to assess sex, ASA classification, the blood transfusion rate, complication rate, 6-month mortality rate, transfer rate, percentage of patients unable to walk at discharge or transfer, and inability to walk 6 months postoperatively. The Spearman’s correlation coefficient by rank test was used to evaluate the correlation between age and GNRI. Statistical significance was set at *p* < 0.05.

## Results

Patients in the normal group (*n* = 62; 14 men and 48 women) were significantly younger at the time of surgery (mean age = 79.6 ± 8.8 years) than those in the undernourished group (mean age = 83.3 ± 6.9 years; *n* = 75; 15 men and 60 women) (*p* < 0.01). The ASA classification, intraoperative blood loss, and surgical time were not significantly different between the groups. The preoperative hemoglobin levels (11.6 ± 1.2 g/dL versus [vs.] 12.8 ± 1.5 g/dL) and postoperative hemoglobin levels (9.0 ± 1.3 g/dL vs. 10.3 ± 1.5 g/dL) were significantly lower in the undernourished group than in the normal group (*p* < 0.01). The blood transfusion rate was significantly higher in the undernourished group than in the normal group (41.3% [31/75] vs. 12.9% [8/62], *p* < 0.01). The preoperative waiting period was longer in the undernourished group than in the normal group (5.5 ± 5.8 days vs. 3.9 ± 2.6 days; *p* < 0.05). The MMSE was longer in the undernourished group than in the normal group (17.9 ± 8.5 vs. 22.7 ± 7.5; *p* < 0.01). A higher percentage of patients in the undernourished group were unable to walk at discharge or transfer (25.3% [19/75] vs. 11.3% [7/62]; *p* < 0.05). The undernourished group contained a higher percentage of patients who required transfers due to the inability to walk (24% [18/75] vs. 9.7% [6/62]; p < 0.05). Furthermore, a higher percentage of patients in the undernourished group were unable to walk after 6 months as was the case at the time of transfer of discharge (41.3% [31/75] vs. 13% [8/62]; *p* < 0.01) (Table 1). The preoperative ADLs is the normal group are as follows: independence 53.2% [33/62], one cane 24.2% [15/62], walker 9.7% [6/62], walking along 12.9% [8/62]. ADLs in the normal group 6 months postoperatively were as follows: independence 29% [18/62], 1 cane 37.1% [23/62], walker 14.5% [9/62], walking along 6.5% [4/62], wheelchair or bedridden 13% [8/62]. On the other hand, in the preoperative undernourished group, ADLs were as follows: independence 29.3% [22/75], 1 cane 22.7% [17/75], walker 14.7% [11/75], walking along 33.3% [25/75]. ADLs in the undernourished group 6 months after surgery are as follows: independence 2.7% [2/75], 1 cane 25.3% [19/75], walker 25.3% [19/75], walking along 5.3% [4/75], wheelchair or bedridden 41.3% [31/75].

**Table 1:** Patients’ characteristics

	Normal group	Undernourished group	*P*-value
Age (years)	79.6 ± 8.8	83.3 ± 6.9	0.009
Sex	Male, 14; Female, 48	Male, 15; Female, 60	0.44
ASA classification	ASA2: 55,	ASA2: 60,	0.24
	ASA3: 7	ASA3: 15	
Preoperative waiting period (days)	3.9 ± 2.6	5.5 ± 5.8	0.03
MMSE	22.7 ± 7.5	17.9 ± 8.5	0.001
Intraoperative blood loss (mL)	169.7 ± 116.8	176.5 ± 111.9	0.71
Surgery time (min)	90.1 ± 25.2	81.4 ± 25.8	0.05
preoperative Hemoglobin levels (g/dL)	12.8 ± 1.5	11.6 ± 1.2	*P* < 0.001
postoperative Hemoglobin levels (g/dL)	10.3 ± 1.5	9.0 ± 1.3	*P* < 0.001
Blood transfusion rate (%)	12.9	41.3	*P* < 0.001
Transfer rate (%)	9.7	24	0.02
Percentage of patients unable to walk at discharge or transfer (%)	11	25.3	0.03
Percentage of patients unable to walk on 6-month (%)	13	41.3	*P* < 0.001
Complication rate (%)	35.5	52	0.04
6-month mortality rate (%)	0	13.3	0.002

*ASA* American Society of Anesthesiologists (ASA), *MMSE* Mini Mental State Examination

The complication rate was significantly higher in the undernourished group than in the normal group (52% [39/75] vs. 35.5% [22/62]; *p* < 0.05) (Table 1). More patients in the undernourished group had pneumonia (Table 2).

**Table 2:** Patients’ complications in the normal group and the undernourished group

Normal group		Undernourished group	
Total	22	Total	39
Pneumonia	3 (13.6%)	Pneumonia	13 (33.3%)
Periprosthetic femoral fracture	5 (22.7%)	Urinary tract infection	6 (15.4%)
Enteritis	3 (7.3%)	Delirium or mental illness	6 (15.4%)
Other	9 (40.1%)	Other	14 (35.9%)

**Fig. 1:**
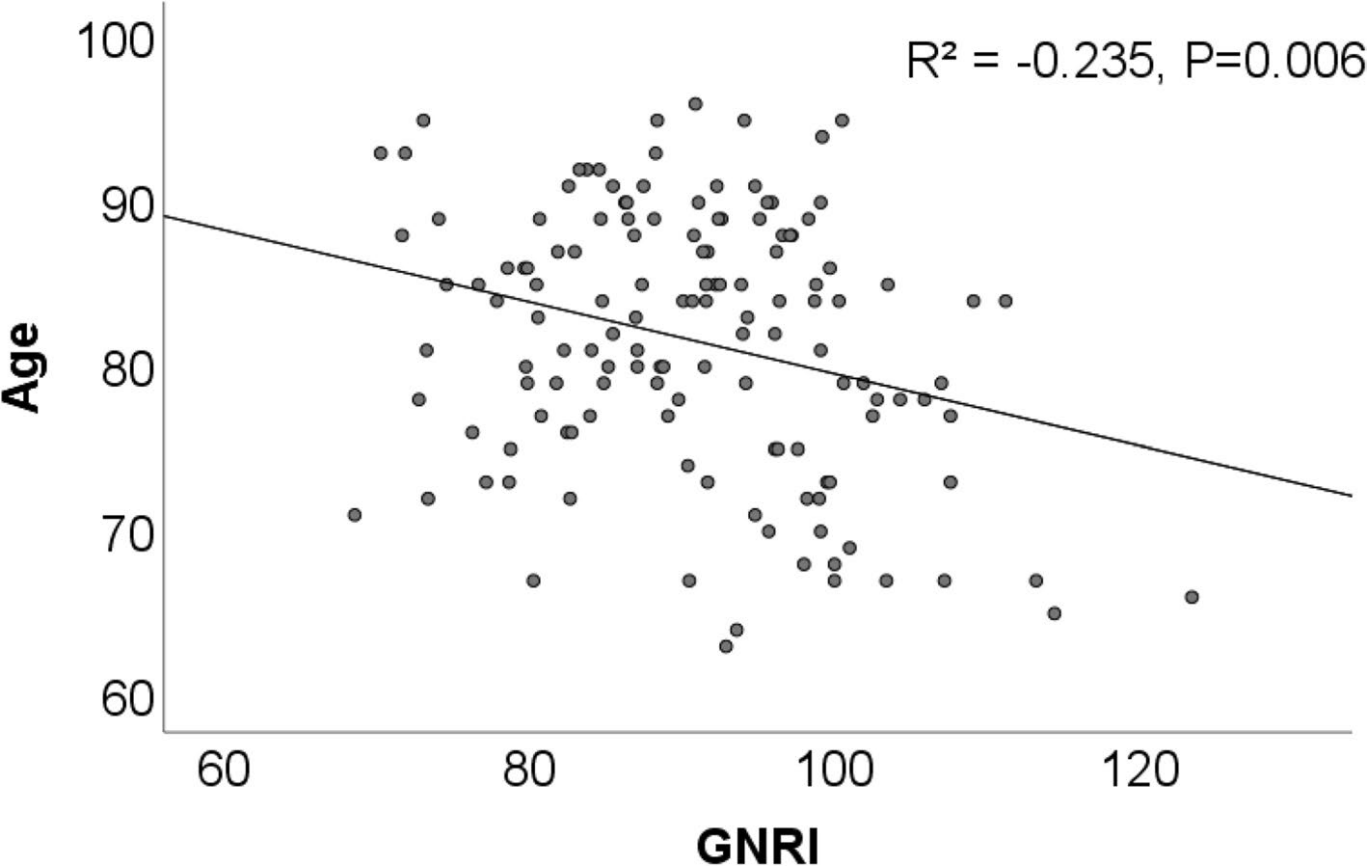
Comparison of the correlation between age and GNRI

The 6-month mortality rate was significantly higher in the undernourished group than in the normal group (13.3% [10/75] vs. 0% [0/62]; *p* < 0.01) (Table 1). Among the 10 patients in the undernourished group who died within 6 months postoperatively, 6 had pneumonia. In addition, a significantly negative correlation was found between age and GNRI (R^2^ = − 0.235. *P* < 0.01) (Fig. 1).

## Discussion

In this study, undernourished patients suffered more postoperative complications, transfers, 6-month mortality rates, and inability to walk 6 months postoperatively.

Fávaro-Moreira NC [14] et al. investigated the risk factors for undernutrition from a systematic review and found that age is also a risk factor for undernutrition because older individuals have a higher prevalence of undernutrition than younger adults. Other reported cases [15, 16] have shown that older individuals have a higher rate of undernutrition compared to their younger counterparts, and are affected by age-related physical, mental, social, and environmental changes. In line to these previous reports, when the correlation between age and nutritional status was evaluated in this study, undernutrition and age were also correlated because GNRI decreased with aging. Therefore, we think that elderly people with lower GNRI than before surgery should be careful because it affects postoperative outcomes.

Approximately 60% of patients with proximal femoral fractures are undernourished, which severely affects postoperative mortality and complications [4, 17, 18]. A weakened immune function resulting from preoperative undernourishment increases the risks of infection and complications and has been associated with increased mortality [19, 20]. Heart failure and pneumonia are the common postoperative complications among patients with proximal femoral fractures. Roche et al. reported that pneumonia was more common than heart failure among patients with proximal femoral fractures [21], which is consistent with the results of the present study.

Dementia is a risk factor for mortality following proximal femoral fracture surgery [2, 22] and it is associated with postoperative infection, delirium, and pneumonia [23]. There are no reports examining the relationships between MMSE and GNRI in previously reported cases; however, some relationships between dementia and nutritional status have been reported. Dementia has been shown to be an independent risk factor for undernutrition, and there is a strong relationship between dementia and undernutrition in elderly patients [14, 22]. In addition, it has been shown that elderly patients with dementia are more likely to have undernutrition due to decreased nutritional status as a result of eating disorders [14, 24]. In this study, the undernutrition group had low MMSE and decreased cognitive function. Considering that cognitive decline may be accompanied by aspiration pneumonia due to eating disorders, we consider that early nutritional intervention including evaluation of swallowing function is desirable from an early stage.

A preoperative scoring system to assess nutritional status has been developed, and this can be used as a reliable indicator to predict postoperative outcomes [25]. In this study, preoperative nutritional status was assessed using the GNRI, which can predict morbidity and mortality among elderly patients [10, 26]. However, there is no consensus on the ideal method for assessing nutritional status in patients with proximal femoral fractures, and a definite assessment method is yet to be established for this specific patient population [22]. The GNRI, which was originally developed to assess patients undergoing hemodialysis [10], is a reliable and valid nutritional index that is also useful for assessing nutritional status in patients with heart failure and stroke [7, 27]. The GNRI was used to assess the nutritional status before surgery for proximal femoral fractures in a recent study [22], and the results showed that undernourished patients had a higher 6-month mortality rate than normal patients, indicating that the GNRI is a useful preoperative predictor of mortality. In the present study, no patients in the normal group died within 6 months postoperatively, and the 6-month mortality rate of patients in the undernourished group was similar to that reported in a previous study [22].

Another previous study reported that preoperatively undernourished patients with proximal femoral fractures exhibited a diminished ability to perform ADLs and had decreased muscle strength at discharge, suggesting that preoperative nutritional status affects the recovery of ADLs in these patients [18]. In addition, low rates of discharge to home have been reported when there is an insufficient recovery of the ability to perform ADLs, which is known to affect the postoperative gait function [9]. The rate of gait recovery following proximal femoral fractures is approximately 50% at 1 year postoperatively, reducing the ability to perform ADLs after surgery. In previous studies, 40% of patients with diminished ADLs were discharged home [4, 5]. In this study, patients in the undernourished group were less likely to be capable of resuming ADLs postoperatively, less likely to be discharged home, and more likely to be transferred to another hospital. In addition, Miu et al. reported that many undernourished proximal femoral fractures patients had insufficient functional recovery even at 6 months, and those who have that poor health condition, multiple existing diseases, and poor self-care ability [15]. In this study, the postoperative ADLs 6 months after patients of femoral neck fractures were evaluated, and the rate of inability to walk was high in the undernourished group, even at 6 months postoperatively. Therefore, it is considered difficult to recover function while being undernourished even at 6 months postoperatively.

In this study, the undernourished group was treated for preoperative pneumonia, and as a result, the preoperative waiting period was longer than that of the normal group. An incidence of 0.3–3.2% has been reported, and this increases the risk of postoperative respiratory failure and the risk of postoperative mortality, which may increase postoperative mortality by up to four times [28, 29]. Preoperative pneumonia, which affects postoperative outcomes, is rarely the focus in studies of proximal femoral fractures because its diagnostic criteria are not clearly stated [29]. Undernutrition is often accompanied by cognitive decline and impaired intake [24]. There is also a risk of aspiration, and such patients may have preoperative pneumonia. In our study, the undernourished group also had high mortality and complication rates, and were more likely to be unable to walk 6 months postoperatively as a result of the extension of the preoperative waiting period due to preoperative treatment for pneumonia, which could lead to poor postoperative outcomes. Preoperative screening using GNRI should be performed on patients with or with dementia, and early nutritional intervention and swallowing evaluation should be performed.

A previous study regarding nutritional status at admission and length of hospital stay reported that undernutrition at admission is associated with longer hospital stays, higher complication rates, and higher mortality rates, suggesting that prolonged hospitalization is associated with postoperative complications related to undernutrition at admission [30]. The awareness of undernutrition in clinical practice is low; therefore, the rate of undernutrition remains underestimated. As a result, undernutrition before undergoing surgery for proximal femoral fractures remains untreated [31]. As undernutrition can deteriorate a patient’s gait function and systemic condition, early nutritional intervention may reduce postoperative complications and mortality and shorten the duration of rehabilitation. Early postoperative nutritional interventions have been reported to reduce mortality and shorten the length of the hospital stay [32, 33]. In this study, undernutrition was associated with a prolonged hospital stay and higher transfer rate, indicating that early preoperative nutritional interventions could help improve patients’ postoperative outcomes.

This study is not without limitations, including its retrospective design and short follow-up period. Long-term outcomes should be assessed in studies with larger sample size. For femoral neck fractures, the invasiveness of surgical treatment differs between osteosynthesis and BHA, and the postoperative results are different. Therefore, we considered this as a different category, and this time we targeted BHA. However, due to the high incidence of trochanteric fractures of the femoral in the elderly [34], we were unable to target patients undergoing osteosynthesis of proximal femoral fractures in this study. In the future, it will be necessary to perform preoperative screening by GNRI as a preoperative evaluation of femoral trochanteric fractures patients and to perform postoperative evaluation by early nutritional intervention. Although the use of the GNRI allowed prediction of postoperative outcomes, the effects of comorbidities (including dementia) were not studied. Furthermore, preoperative cognitive function evaluation was possible, but the effect of nutritional intervention on undernourished patients with preoperative cognitive decline in the postoperative course could not be evaluated. Another limitation of this study is that data regarding postoperative nutritional interventions were not standardized. Therefore, the effects of postoperative nutritional interventions on postoperative outcomes cannot be determined. In addition, muscle strength and other aspects of physical function should be assessed in future studies to determine how the preoperative nutritional status affects improvement in functional outcomes. Preoperative nutritional screening should be performed to determine whether early and effective nutritional interventions and rehabilitation can help restore the ability of undernourished patients to perform ADLs.

## Conclusions

Nutritional status before femoral neck fracture surgery affects postoperative complications, mortality, and gait function. The GNRI is a simple screening tool that can be used at admission to effectively predict these outcomes. This study suggests that the GNRI may be useful for predicting not only postoperative complications and mortality but also gait function recovery after femoral neck fracture surgery.

## Data Availability

All data generated or analyzed within this study are included in this published article.

## Abbreviations

GNRI: Geriatric Nutritional Risk Index
ADL: Activity of daily living
BHA: Bipolar hemiarthroplasty
vs.: Versus
MMSE: Mini Mental State Examination
ASA: American Society of Anesthesiologists Physical Status
